# Refugee children and adolescents in Greece: two case reports

**DOI:** 10.1080/20008198.2017.1351179

**Published:** 2017-09-29

**Authors:** Ignatia Farmakopoulou, Kalliopi Triantafyllou, Gerasimos Kolaitis

**Affiliations:** ^a^ Department of Child Psychiatry, Medical School, National and Kapodistrian University of Athens, ‘Aghia Sophia’ Children’s Hospital, Athens, Greece

**Keywords:** Refugee, trauma, post-traumatic stress disorder, children and adolescents

## Abstract

In 2015, in the EU-28 (total population 508 million inhabitants), the number of first-time asylum applicants was 1,255,688; 29% of them were minors (19% under 14 years and 10% between 14 and 17 years). In Greece, more than 710,000 refugees, mainly from Syria (69%) and Afghanistan (21%), arrived by boat in 2015 (Hebebrand et al., 2016; UNHCR, 2016). It is well documented that young refugees have a high prevalence of physical disorders and nutritional deficiencies and are consequently in need of provision of somatic health care. At the same time, as they have been exposed to many risks before, during their journey and upon arrival to the host country, they are vulnerable to developing post-traumatic stress disorder (PTSD), anxiety, mood, externalizing, and other psychiatric disorders. According to some studies, PTSD seems to be related mainly, but not exclusively, to pre-migration experiences, and depression to post-migration stressors such as unsafe asylum status, etc.

During the last few years we have encountered increasing numbers of referred refugee youths in outpatients and emergencies at the ‘Aghia Sophia’ Children’s Hospital (one of the biggest in Europe) (Farmakopoulou, Liakopoulou, Hantzara, & Kolaitis, 2012). These children are presenting with severe new-onset symptoms related to trauma, e.g. PTSD symptoms, conduct and sleeping problems, and/or pre-existing problems, e.g. autism spectrum disorders, psychosis, etc. In some cases there are suspicions of physical and sexual abuse at reception sites. The management of such cases becomes even more difficult because of communication difficulties due to language barriers.

The current report focuses on two youths’ cases who are refugees from two different countries (Syria and Afghanistan). S. is a nine-year-old boy from Syria, who had a serious accident at the borders of Greece and Turkey; he had third-degree burns on most of his body caused by an electric shock from an abandoned train wagon, and he was hospitalized in the paediatric hospital. For over two months he was in a critical medical condition because of septicaemia, he suffered acute pain, and every two weeks had a series of surgeries (skin transplantations). He was in an isolated chamber and felt extremely lonely and bored. He had nightmares, symptoms of depression, challenging behaviour, and for a short period he had illusions (he used to see an unknown man who was dressed in white and nodded at him). His treatment costed thousands of euros, and the medical and nursing staff did their best to help him. Initially, before the psychiatric and counselling intervention, the medical and nursing staff faced a series of difficulties with the boy and his family since the main aim of his parents was to move to west Europe, therefore their cooperation was quite difficult. The boy was finally transferred to the country that his family had planned to move to.

M. is a 14-year-old unaccompanied minor from Afghanistan. His mother and his other two siblings remained in Afghanistan, whereas his father was in prison in Turkey. He had destructive anger outbursts, oppositional and challenging behaviour, self-harming behaviour (cutting himself with knives), intense nightmares, and separation anxiety difficulties. He attended a refugee shelter and when at a crisis he would be examined by mental health staff at the emergency departments of various paediatric hospitals. Communication difficulties often made it difficult for the staff to provide psychotherapy. For this reason, translators were involved on a regular basis. He had, and still has, a great wish to migrate to Germany, since he believes that refuges are very well treated in that country.

These cases illustrate that the exposure of young refugees to violent events is associated with a range of psychosocial problems and that multidisciplinary approaches are needed. The Greek mental health and social services have made substantial efforts to face the refugee mental health and social problems and to offer help to families and especially to unaccompanied minors who face multiple traumatizations through deliberate separation from their own families (Farmakopoulou, Liakopoulou, Hantzara, & Kolaitis, 2012). The European Society of Child and Adolescent Psychiatry has called inter alia for all basic health care to be provided to migrants, focusing on youths’ physical and mental health (Hebebrand et al., 2016).
